# MiR-126 negatively regulates PLK-4 to impact the development of hepatocellular carcinoma via ATR/CHEK1 pathway

**DOI:** 10.1038/s41419-018-1020-0

**Published:** 2018-10-12

**Authors:** Jie Bao, Yan Yu, Jianan Chen, Yuting He, Xiaolong Chen, Zhigang Ren, Chen Xue, Liwen Liu, Qiuyue Hu, Juan Li, Guangying Cui, Ranran Sun

**Affiliations:** 1 Key Laboratory of Clinical Medicine, the First Affiliated Hospital of Zhengzhou University, Zhengzhou, 450052 China; 2 Precision Medicine Center, the First Affiliated Hospital of Zhengzhou University, Zhengzhou, 450052 China; 3 National Engineering Laboratory for Internet Medical System and Application, The First Affiliated Hospital of Zhengzhou University, Zhengzhou, 450052 Henan China

## Abstract

Emerging evidence has shown that microRNA-126 (miR-126) is aberrantly downregulated and plays a vital role in carcinogenesis in various cancers, including HCC. However, the underlying biological mechanisms of miR-126 in HCC are still largely unknown. In present study, we found that miR-126 was downregulated both in HCC tissues and cell lines. Low expression level of miR-126 was associated with poor overall survival (OS), late TNM stage and the presence of recurrence. Overexpression of miR-126 significantly decreased cell proliferation, metastasis and promoted apoptosis in vitro. Additional, high miR-126 expression reduced the tumor growth in vivo. Further we discovered that PLK (polo-like kinases)-4, a critical regulator in cell cycle, was a target of miR-126. PLK-4 overexpression could rescue the inhibitory effects of miR-126 on cell proliferation and invasion. Moreover, PLK-4 mRNA and protein levels were significantly upregulated in HCC tissues and positively associated with malignancies and poor OS. Knockdown PLK-4 significantly inhibited cell proliferation, invasion and promoted cell apoptosis in vitro whereas decreased tumor growth in vivo. More importantly, bioinformatics analysis combined with validation experiments in vitro and in vivo showed that activation of the ATR/CHEK1 pathway was involved in the oncogenic functions of PLK4 in HCC. We also validated that PLK4 could directly interact with ATR through CoIP assay. Taken together, we demonstrate that miRNA-126/PLK-4 axis is critical for tumorigenesis and progression of HCC, and the newly identified PLK-4/ATR/CHEK1 pathway may be a potential therapeutic target for HCC treatment.

## Introduction

Hepatocellular carcinoma (HCC), one of the most common primary liver cancers, represents the third leading cause of cancer-related death worldwide with high incidence and post-surgical recurrence^1,2^. Most HCC patients are diagnosed at late stages due in part to lack of effective early diagnostic biomarkers and have a very poor 5-year survival despite treatment^3,4^. Therefore, it is urgent and important to explore the molecular mechanism underlying HCC progression, and find novel biomarkers and therapeutic targets for HCC.

miRNAs are small single-strand non-coding RNAs that regulate gene expression through binding to the 3′-untranslated region (UTR) of target mRNAs^5^. Accumulating evidence indicates that miRNAs are involved in a wide range of physiological and pathological processes, including proliferation, differentiation, metabolism and apoptosis^6,7^. Additionally, numerous studies have revealed that miRNAs are dysregulated in various types of cancer and contribute to tumorigenesis, including HCC^8–10^. MiR-126, as one of miRNAs, has been widely found to be downregulated and may function as a tumor suppressor in gastric cancer^11^, lung cancer^12^, prostate cancer^13^ and HCC^14^. However, the underlying molecular mechanisms of miR-126 in HCC are still not well characterized.

Polo-like kinase (PLK) proteins are characterized by a highly conserved Nterminal serine/threonine kinase domain and one or two polo boxes in the C-terminal region, which are crucial for subcellular localization, binding of specific phosphopeptides, and centriole duplication^15,16^. PLK-4, a number of PLKs family, plays an important role in the regulation of centriole duplication^17^, and carcinogenesis^18^. Overexpression of PLK-4 has been reported for a variety of neoplasms^19,20^, indicating its oncogenic role in cancer progression. However, the biological functions of PLK-4 and its molecular mechanisms in HCC remain unclear.

Ataxia telangiectasia and Rad3-related (ATR)-checkpoint kinase 1 (CHEK1) signaling is critical for genomic stability through regulating DNA damage response (DDR)^21,22^. Accumulating evidence suggests that ATR/CHK1 pathway is upregulated in various types of cancer and promote tumor progression, such as breast cancer^23^, oral squamous cell carcinoma^24^ and pancreatic cancer^25^, through regulating cell cycle and DNA damage response (DDR). However, the ATR/CHK1 signaling activity and functional roles in HCC are still unknown.

In this study, we showed that miR-126 was dramatically downregulated in HCC tissues and acted as a tumor suppressor. In addition, we confirmed that PLK-4 was a target of miR-126 and upregulated PLK-4 was markedly associated with poor prognosis in HCC patients. PLK-4 silencing inhibited cell proliferation, metastasis and tumor growth in vitro and in vivo, and promoted apoptosis. Moreover, overexpression of miR-126 inhibited HCC cells proliferation, but increasing PLK-4 expression reversed this change. Mechanistically, we found that there was a positive correlation between PLK-4 expression and ATR/CHEK1 pathway activation. Taken together, we demonstrate that miRNA-126/PLK-4 axis is critical for tumorigenesis and progression of HCC, and the newly identified PLK-4/ATR/CHEK1 pathway may be a potential therapeutic target for HCC treatment.

## Results

### MiR-126 is downregulated in HCC tissues and cells as well as negatively correlated with clinic-pathologic parameters

**Fig. 1:**
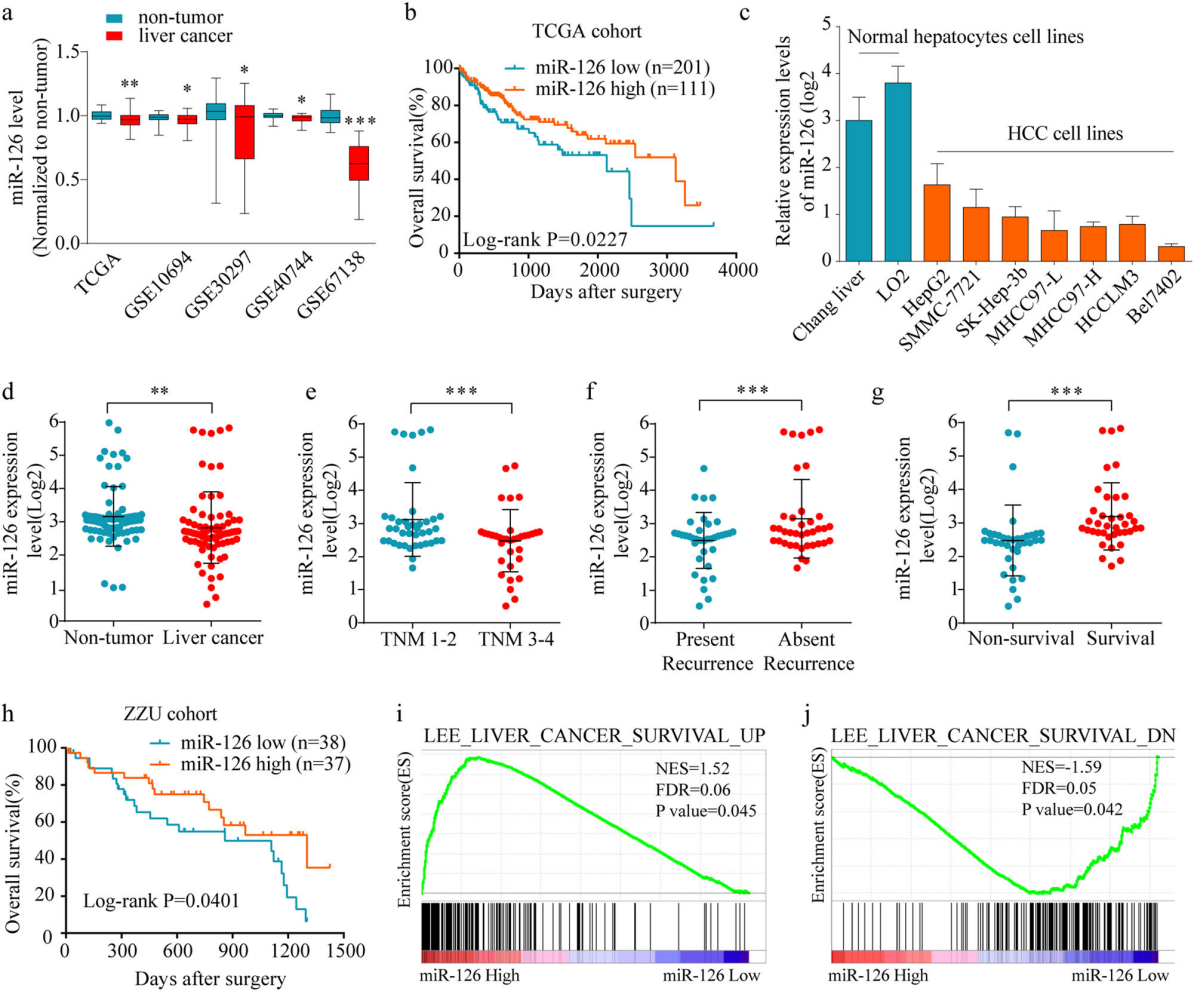
The expression level of miR-126 in HCC tissues and cell lines. **a** The expression level of miR-126 in liver cancer and non-tumor tissues from TCGA and GEO database. **b** Kaplan–Meier analysis of overall survival of 312 liver cancer patients from TCGA-LIHC cohort based on miR-126 expression. **c** Comparison of the expression level of miR-126 in normal liver cells and HCC cell lines. **d** The expression level of miR-126 in liver cancer and non-tumor tissues from ZZU cohort 1. **e** The inverse correlation of miR-126 expression level with TNM classification. (**f**), recurrence (**g**), patient survival rate (**h**), and overall survival days after surgery. (**i**). The Gene Set Enrichment Analysis plot indicated a significant correlation between miR-126 expression and the genes highly expressed in hepatocellular carcinomas with a good survival prognosis (LEE_LIVER_CANCER_SURVIVAL_UP); (**j**) and a poor survival prognosis (LEE_LIVER_CANCER_SURVIVAL_DN). Mean ± SD, unpaired Student’s *t* test or Kaplan–Meier analysis

To confirm the expression of miR-126 in HCC, we firstly analyzed the HCC miRNA expression profile data from TCGA and GEO database. The results showed that miR-126 was significantly downregulated in HCC tissues compared with non-tumor liver tissues (Fig. 1a). Moreover, we found that the patients with high miR-126 expression exhibited a better overall survival rate than those with low miR-126 expression (Fig. 1b). Then we examined the miR-126 expression level in normal hepatocyte cell line and HCC cell lines. We observed that miR-126 was significantly downregulated in HCC cell lines (Fig. 1c). Meanwhile, we assessed its expression in a 75 pairs of HCC and adjacent non-tumorous tissues (ZZU cohort 1) using qPCR. Consistent with TCGA results, the expression of miR-126 was significantly downregulated in ZZU cohort 1 (Fig. 1d). Low miR-126 expression was dramatically associated with late tumor stage (Fig. 1e) and the presence of recurrence (Fig. 1f). Additionally, miR-126 expression was inversely associated with non-survival cases (Fig. 1g) and overall survival rate after surgery (Fig. 1h). Furthermore, the Gene Set Enrichment Analysis (GSEA) of the TCGA-LIHC data set revealed a high expression level of miR-126 associated with gene signatures of better survival (Fig. 1i, j).

A detail summary of the relationship between miR-126 expression and clinico-pathological features of TCGA-LIHC cohort and ZZU cohort 1 was shown in Supplementary Table 1 and Table 2, respectively. These findings suggested that miR-126 may act as a tumor suppressor in HCC.

### Over-expression of miR-126 suppress the tumor cell proliferation and metastasis in vitro

**Fig. 2:**
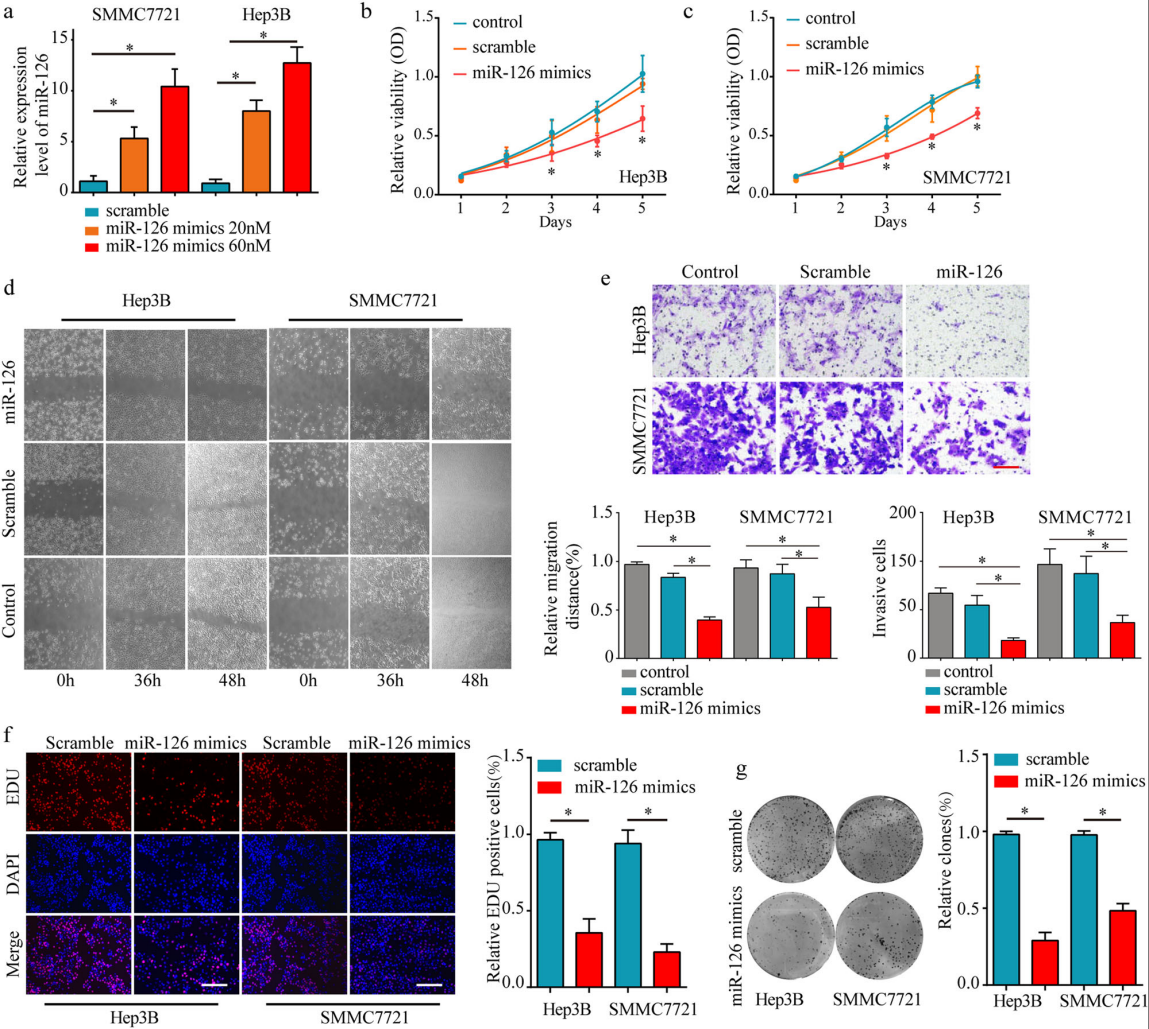
Over-expression of miR-126 suppress the tumor cell proliferation and metastasis in vitro. Transfection of the scramble and miR-126 mimics into two HCC cell lines, Hep3B and SMMC-7721, increased the expression level of miR-126 in a dose-dependent manner (**a**). Cell viability assay of Hep3B (**b**) and SMMC-7721 (**c**) after transfection of scramble and miR-126 mimics compared with control cells. **d** Representative images of scratch assay of Hep-3b and SMMC-7721 in 0 h, 36 h, 48 h after transfection scramble and miR-126 mimics. Scale bars, 50 μm. **e** Transmembrane invasion assay of Hep-3b and SMMC-7721 after transfection of scramble and miR-126 mimics. Scale bars, 200 μm. **f** EDU staining assay of Hep-3b and SMMC7721 after transfection scramble and miR-126 mimics was performed. Scale bars, 50 μm. **g** Colony formation assay of Hep-3b and SMMC7721 after transfection scramble and miR-126 mimics was performed. Mean ± SD, *P < 0.05, unpaired Student’s *t* test or one-way ANNOVA followed by multiple *t* test

To investigate the function of miR-126 on HCC cells, miR-126 mimics and scramble were transfected into two HCC cell lines, Hep3B and SMMC7721. Transfection efficiency was confirmed by real-time PCR analysis (Fig. 2a). MTT assay showed that the cell viability was significantly decreased after transfected with miR-126 mimics compared with control group (Fig. 2b, c). Wound healing assay and invasion assay confirmed that overexpression of miR-126 reduced HCC cell migration and invasion capacity (Fig. 2d, e). In addition, miR-126 overexpressing inhibited the cell proliferation and colony formation of HCC cells by EDU staining and colony formation assay (Fig. 2f, g). Taken together, these data indicate that miR-126 negatively regulates HCC cell proliferation and metastasis in vitro.

### PLK-4 is a direct functional target of miRNA-126

**Fig. 3:**
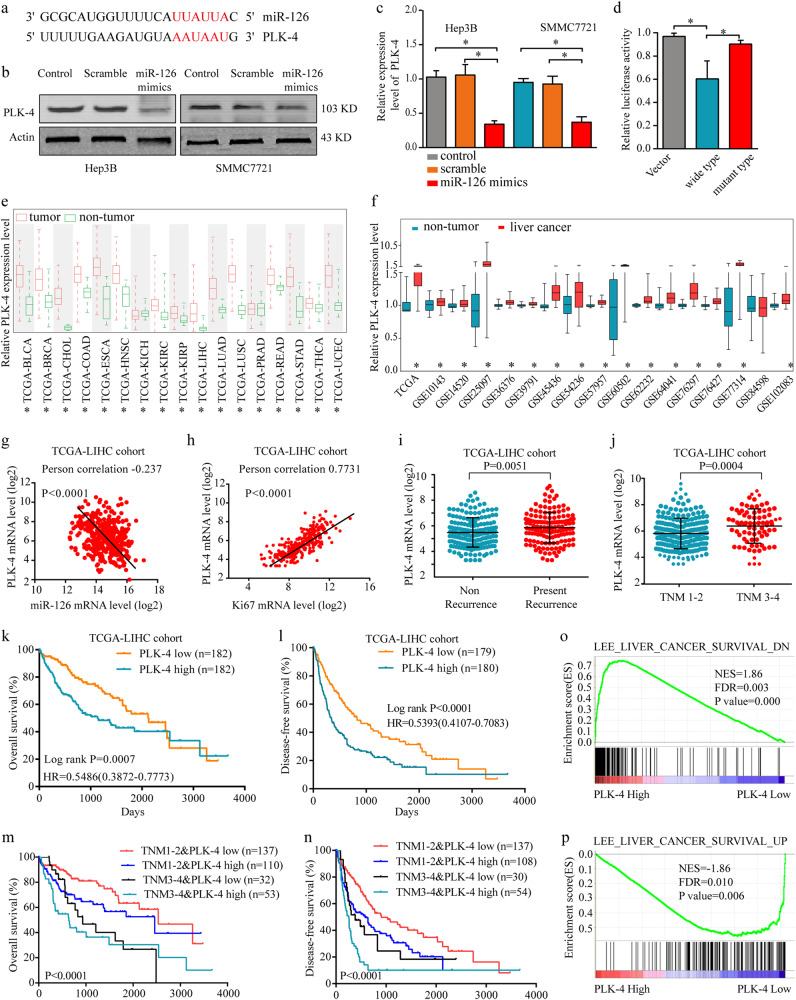
Identification of PLK-4 as a miR-126 target in HCC. **a** PLK-4 commonly was found as the effector of miR-126 based on the 3′ untranslated regions of PLK-4. **b** The expression of PLK-4 were detected in HCC cell lines after miR-126 mimics transfection by western blot. **c** The expression of PLK-4 was detected in HCC cell lines after miR-126 mimics transfection by RT-PCR. **d** Overexpression of miRNA-126 decreased PLK-4 3′-UTR dependent luciferase activity. Cells were cotransfected with either miRNA-126 mimics or PLK-4 3′-UTR reporter wild-type (WT) or mutant (MT) plasmid. The dual luciferase activity was measured by illuminometer. **e** The expression level of PLK-4 in Pan-cancer and non-tumor tissues from TCGA database. **f** The expression level of PLK-4 in liver cancer and non-tumor tissues from TCGA and GEO database. **g** The negative correlation between miR-126 and PLK4 expressions. **h** The positive correlation of PLK-4 levels with Ki67 from TCGA-LIHC cohort database was analyzed. **i** Comparison of the expression level of PLK-4 levels in non-recurrence patients with that in present-recurrence patients. **j** Comparison of the expression level of PLK-4 levels in different clinical stages. **k**, **l** OS analysis and DFS analysis of the patients based on PLK-4 expression in TCGA-LIHC cohort. Mean ± SD, unpaired Student’s *t* test or Kaplan–Meier analysis. The HCC gene-expression data set from TCGA was sorted into PLK-4 low and high expression groups. **m**, **n** Stratified analyses between TNM stages and PLK-4 expression on OS and DFS. **o**, **p** The Gene Set Enrichment Analysis plot indicated a significant correlation between PLK-4 expression and the genes highly expressed in HCC with a good survival prognosis (LEE_LIVER_CANCER_SURVIVAL_UP) and a poor survival prognosis (LEE_LIVER_CANCER_SURVIVAL_DN). Mean ± SD, unpaired Student’s *t* test or Kaplan–Meier analysis

MicroRNAs can serve as gene expression regulators by binding to the 3′ untranslated regions of their target mRNAs^26^. In order to identify the target genes of miR-126, we searched for candidate genes by 2 bioinformatics algorithms: TargetScan 6.2 (http://www.targetscan.org/) and miRBase (http://www.mirbase.org/) micoRNA databases. From both algorithms, PLK-4 was commonly found as the effector of miR-126 based on the 3′ untranslated regions of PLK-4 (Fig. 3a). To confirm the miR-126 could target PLK-4 gene, qRT-PCR analysis and western blot were performed in the miR-126 overexpressing HCC cell lines and in control cells. We found that PLK-4 significantly downregulated in the miR-126-overexpressing cell lines (Fig. 3b, c). To further confirm miR-126 could directly bind to PLK-4, Luciferase reporter assay was conducted and we found that luciferase activity of the wild-type reporter, but not the mutant, was significantly decreased when transfected with miRNA-126 mimics, proving the specificity of the interaction between miRNA-126 and the PLK-4 mRNA 3′UTR (Fig. 3d).

### PLK-4 is upregulated and associated with poor prognosis in HCC

To investigate the expression of PLK-4 in HCC, we analyzed the expression profiles data from TCGA and GEO database. The results demonstrated that PLK-4 expression was commonly upregulated in TCGA Pan-cancer compared with non-tumor tissues, especially in HCC (Fig. 3e, f). Meanwhile, PLK-4 expression level was negatively associated with miR-126 expression (*R* = −0.237, *P* < 0.0001) (Fig. 3g) and positively related to the expression of Ki67, a proliferation marker of HCC cells^27^ (*R* = 0.7731, *P* < 0.0001) (Fig. 3h). Moreover, the patients with a high PLK-4 expression exhibited a higher recurrence and TNM stage (Fig. 3i, j), whereas exhibited a poor overall survival and disease-free survival rate (Fig. 3k, l), regardless the state of TNM stage (Fig. 3m, n). Consistently, the GSEA revealed a high expression level of PLK4 correlated with gene signatures of poor survival (Fig. 3o, p).

**Fig. 4:**
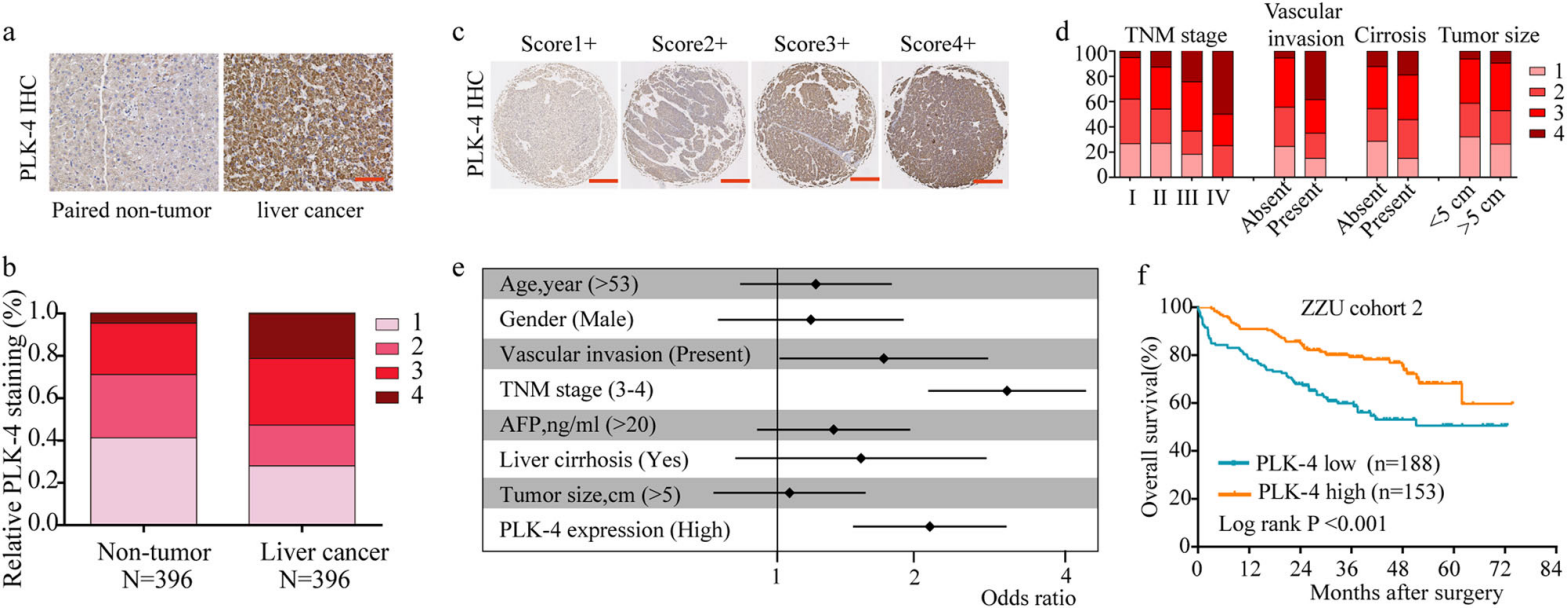
PLK-4 expression is positive associated with poor prognosis of HCC. **a**, **b** PLK-4 expression was significantly increased in HCC tissues compared with non-tumor tissues by IHC assay and based on PLK4 staining score, the PLK4 expression was analyzed in HCC tissues compared with that in non-tumor tissues. Scale bars = 100 μm. **c** Representative pictures of PLK-4 expression in HCC tissues. Scale bars = 500 μm. **d** The detailed staining scores of PLK-4 in TNM stage, vascular invasion, liver cirrhosis and tumor size. **e** Univaraite analysis between clinicopathologic features and OS in HCC patients. Mean ± SD, *P < 0.05, **P < 0.01, unpaired Student’s *t* test or one-way ANNOVA followed by multiple *t* test. **f** OS analysis of the patients based on PLK-4 expression in ZZU cohort 2. Mean ± SD, unpaired Student’s *t* test or Kaplan–Meier analysis

To further address the protein changes of PLK-4 in HCC, immunohistochemical analysis (IHC) was performed in tissue microarray (TMA) containing 396 tissues of HCC patients (ZZU cohort 2). Consistently, we found that PLK-4 protein expression was also significantly upregulated in HCC tissues and positively associated with advanced TNM stage and the presence of vascular invasion (Fig. 4a-d). Kaplan–Meier analysis showed that OS was shorter in HCC patients with higher PLK-4 expression (Fig. 4f). A detail summary of the relationship between PLK-4 expression and clinico-pathological features of HCC was shown in Table 1. Univariate and multivariate analysis disclosed that TNM stage, vascular invasion and PLK-4 expression were significant correlated with OS in HCC patients (Fig. 4e, Table 2). Collectively, these data indicated that high PLK-4 expression could be considered as a prognosis marker and might play an important role in HCC progression.

**Table 1:** Association of PLK-4 expression and clinical features in ZZU cohort 2

Clinicopathological features		PLK-4 expression		*χ* ^2^	*P* value	Survival		*χ* ^2^	*P* value
		High (*n* = 153)	Low (*n* = 188)			Live (*n* = 231)	Dead (*n* = 110)		
Age (years)	≥53	69	100	2.210	0.137	120	49	1.633	0.201
	<53	84	88			111	61		
Gender	Male	117	148	0.247	0.619	178	87	0.178	0.673
	Female	36	40			53	23		
Vascular invasion	Absent	134	168	0.412	0.521	210	92	4.135	**0.042**
	Present	18	18			19	17		
TNM stage	I and II	105	162	15.278	**<0.001**	197	70	20.546	**<0.001**
	III and IV	48	26			34	40		
AFP	≤20	77	89	0.301	0.583	120	46	3.061	0.080
	>20	76	99			111	64		
Cirrhosis	Absent	14	12	0.917	0.338	15	11	1.301	0.254
	Present	139	176			216	99		
Tumor volume	<5 cm	82	108	0.507	0.476	127	63	0.159	0.690
	≥5 cm	71	80			104	47		
PLK-4 expression	High	—	—		—	88	65	13.279	**<0.001**
	Low	—	—		—	143	45		

Bold values indicate statistical significance, *P* < 0.05

**Table 2:** Independent prognostic factors for OS by multivariate analyses in ZZU HCC cohort 2

Univariate analysis	Relative risk	95% CI	*P* value
TNM (III and IV vs. I and II)	2.692	1.750–4.139	**<0.001**
Vascular invasion (Present vs. Absent)	1.081	0.619–1.888	**0.042**
PLK-4 expression (High vs. Low)	1.877	1.267–2.780	**0.002**

Bold values indicate statistical significance, *P* < 0.05

### PLK-4′s functions in tumor proliferation and apoptosis are opposite to those of miR-126 in HCC

**Fig. 5:**
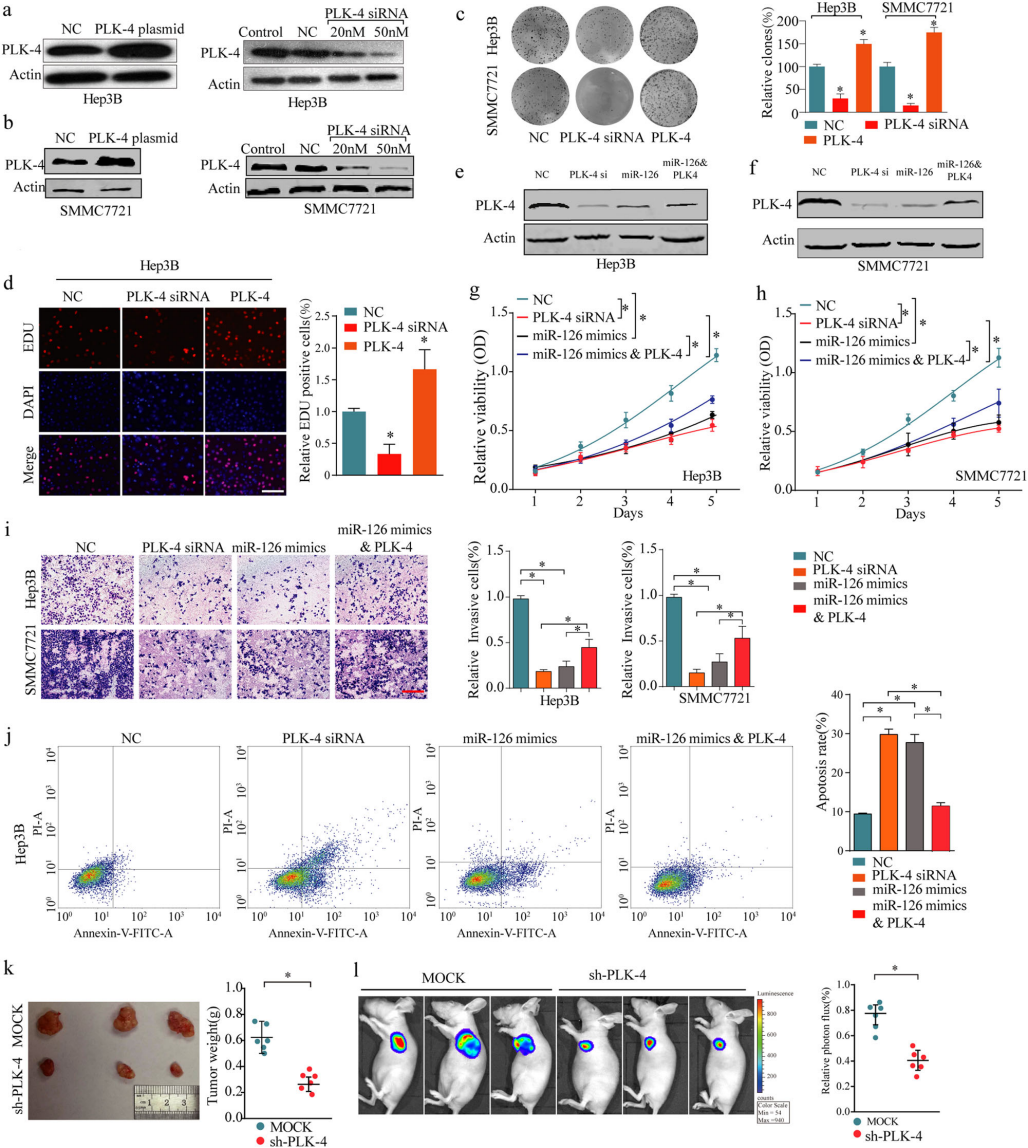
In vitro and in vivo functional analysis of miR-126/PLK-4 axis in HCC. **a**, **b** Western blotting analysis of PLK-4 expression in HCC cells after transfected with PLK-4 plasmid or PLK-4 siRNA. **c** Knockdown of PLK-4 dramatically inhibited the cell colony formation ability. **d** Overexpression of PLK-4 promoted cell growth in Hep-3B cells as evaluated by EDU assay. Scale bars, 50 μm. **e**, **f** The expression levels of PLK-4 after transfected with PLK-4 siRNA, miR-126 mimic, and miR-126 mimic&PLK-4. **g**, **h** PLK-4 knockdown suppressed cell proliferation, while overexpression of PLK-4 abolished the suppressive effect of miR-126 on cell proliferation rate in liver cancer cells, as determined by the MTT assays. **i** Decreased PLK-4 inhibited cell invasion, while overexpression of PLK-4 abolished the suppressive effect of miR-126 on cell invasion ability in HCC cells. Scale bars, 50 μm. **j** The rate of cell apoptosis was much higher after transfection with PLK-4 siRNA or miR-126 mimics, but was attenued in the group of co-transfected with miR-126 mimics and PLK-4 plasmid. **k**, **l** The in vivo effect of PLK-4 was evaluated in xenograft mouse models bearing tumors originating from SMMC-7721 cells; *n* = 6 per group. Mean ± SD, *P < 0.05, **P < 0.01, unpaired Student’s *t* test or one-way ANNOVA followed by multiple *t* test

To explore the functional role of PLK-4 in HCC progression, we transfected HCC cells with PLK-4 plasmid or PLK-4 siRNA. The Transfection and expression efficiency were confirmed by western blot (Fig. 5a, b). The colony formation and EDU assays showed that the ability of cell proliferation was inhibited after PLK-4 knockdown but was promoted after PLK-4 overexpression (Fig. 5c, d). As shown in Fig.5e, f, the expression of PLK4 was decreased after transfected with PLK4 siRNA or miR-126 mimic, while could be counteracted by cotransfected with miR-126 mimic and PLK4. MTT assays presented that PLK-4 silencing significantly reduced the viability of HCC cells while overexpression of PLK-4 abolished the suppressive effect of miR-126 on cell proliferation rate in HCC cells (Fig. 5g, h). The results of transwell assay showed that PLK-4 knockdown and miR-126 overexpressing significantly reduced the ability of cell invasion, whereas cells co-transfected with miR-126 mimics and PLK-4 plasmid could counteract the suppressive effect (Fig. 5i). Furthermore, the rate of cell apoptosis was much higher after transfection with PLK-4 siRNA or miR-126 mimics, but was reversed in the group of co-transfected with miR-126 mimics and PLK-4 plasmid (Fig. 5j). Additionally, in vivo experiments, after injection with sh-PLK-4 and MOCK into the nude BALB/c male mice respectively, the growth rate and tumor weight were markedly less after PLK-4 knockdown (Fig. 5k, l). Taken together, these results demonstrated that miR-126 played its anti-proliferative role, at least in part, through regulating PLK-4 in HCC.

### Activation of the ATR/CHEK1 pathway was involved in the oncogenic functions of PLK4 in HCC

**Fig. 6:**
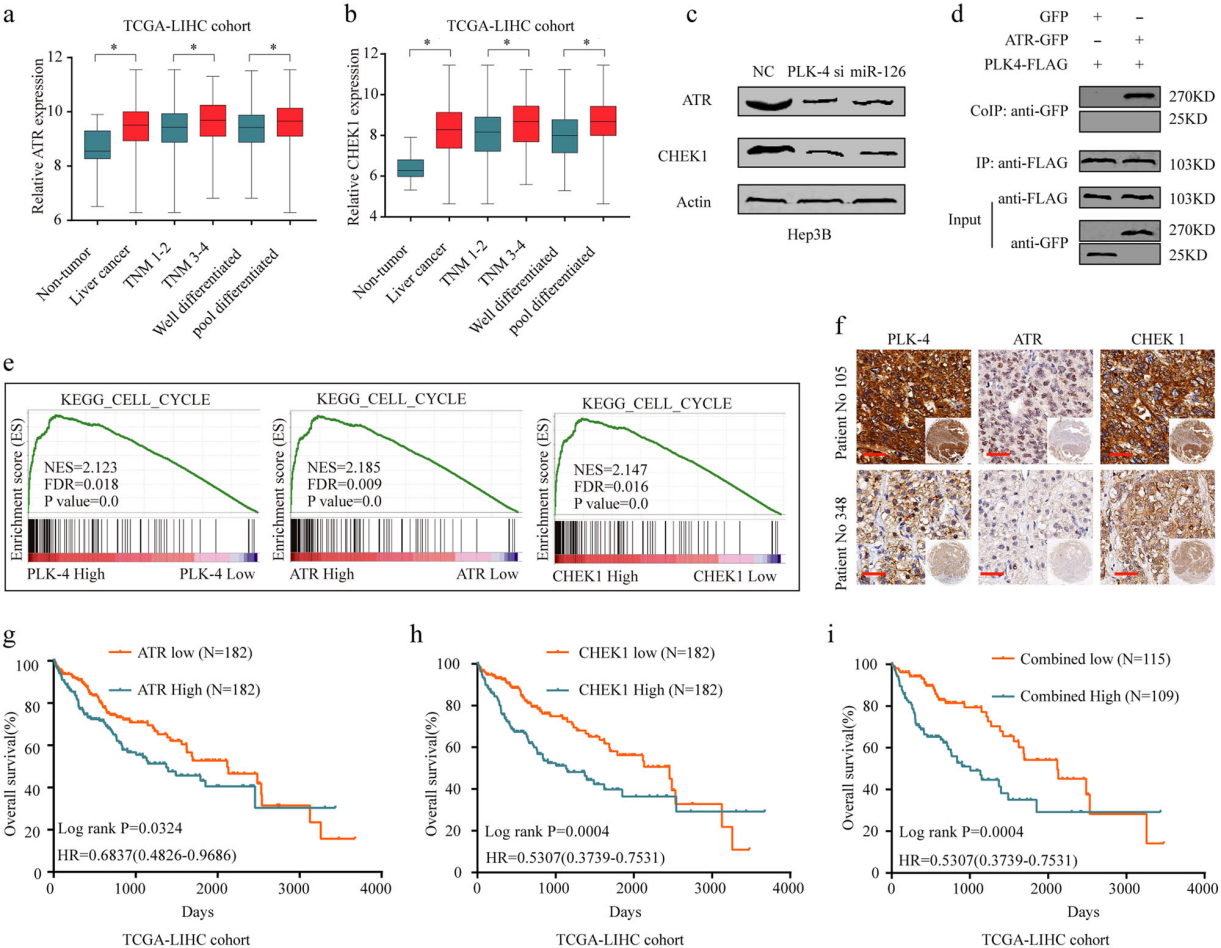
Activation of the ATR/CHEK1 pathway was involved in the oncogenic functions of PLK4 in HCC. **a**, **b** The expression levels of ATR and CHEK1 in TCGA-LIHC data set. **c** PLK-4 silencing causes a decrease in the levels of ATR and CHEK1 expression in HCC cells. **d** Expression and CoIP of PLK4-FLAG and ATR-GFP. Hep3B cells expressed PLK4-FLAG and GFP (left panels) were used as negative controls. The PLK4-FLAG protein was immunoprecipitated with anti-FLAG antibody, and the presence of ATR-GFP or GFP protein was detected by immunoblot analysis with anti-GFP antibody. These experiments were repeated three times with similar results. **e** The Gene Set Enrichment Analysis plot indicated a significant correlation between PLK-4, ATR and CHEK1 expression and cell cycle. **f** IHC staining showed the consistent expression pattern of PLK-4, ATR and CHEK1 in HCC tissues. Scale bars = 100 μm. **g**–**i** High ATR and CHEK1 expression were significantly associated with poor prognosis in HCC patients. Mean ± SD, *P < 0.05, **P < 0.01, unpaired Student’s *t* test or Kaplan–Meier analysis

To further elucidate the molecular mechanisms underlying functional role of PLK-4 in HCC, bioinformatics analysis was conducted based on TCGA-LIHC cohort. We found CHEK1 and ATR were significantly upregulated in HCC tissues and positively correlated with advanced TNM stage and poor differentiation (Fig. 6a, b). The pearson correlation analysis demonstrated that there were significantly positive correlation among PLK-4, ATR and CHEK1 expression **(**Supplementary Fig. 1a-c). Moreover, both ATR and CHEK1 expression were positively related to Ki67 expression **(**Supplementary Fig. 1d, e**)**. Furthermore, western blot confirmed that the protein levels, of ATR and CHEK1, respectively, were drastically reduced in PLK-4 silenced cells (Fig. 6c). To determine the interaction of PLK4 and ATR, we next performed coimmunoprecipitation (CoIP) assays in Hep3B cells. We extracted total protein and immunoprecipitated PLK4 using anti-FLAG antibody and then detected the presence or absence of ATR with anti-GFP antibody. The results indicated that PLK4 could interact with ATR **(**Fig. 6d**)**. The GSEA results identified high expression of PLK4, ATR and CHEK1 exhibited consistent gene signatures of cell cycle **(**Fig. 6e**)**, DNA replication and mismatch repair **(**Supplementary Fig. 2**)**. Additionally, the results of IHC staining showed that both ATR and CHEK1 expression were upregulated in PLK-4 high expression HCC tissues (Fig. 6f). More interestingly, we found that high ATR and CHEK1 expression indicated poor OS and DFS for HCC patients from TCGA LIHC database (Fig. 6g-i, Supplementary Fig. 1f, g). Overall, our results demonstrate that PLK-4 function as an oncogene through activating ATR/CHEK1 pathway in HCC.

### miR-126 suppresses tumor proliferation in vivo through regulating PLK-4/ATR/CHEK1 pathway

**Fig. 7:**
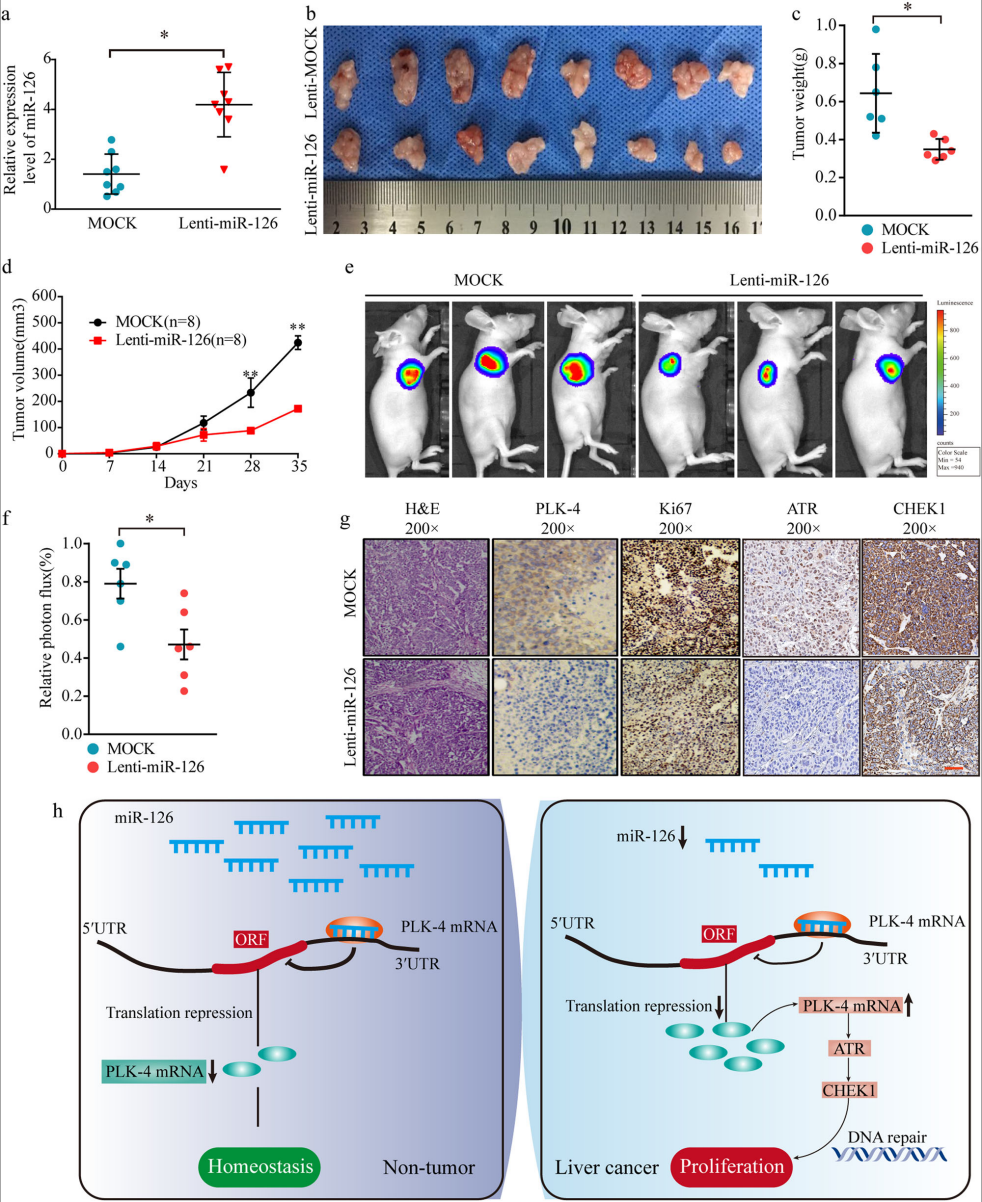
In vivo functional analysis of miR-126 in HCC. **a** Confirmation of miR-126 expression level in mock and miR-126 infected SMMC-7721 cells. Representative tumor images in a xenograft tumor mice model using the SMMC-7721 stable cell line expressing miR-126. **b**. Comparison of tumor weight (**c**) and volume (**d**) in mock and miR-126 infected SMMC-7721 cells. **e** Representative photographs of the xenograft tumor mice model at 4 weeks after the injection of HCC cells were taken by an IVIS system. **f** The strength of the luciferase signal of xenograft tumor was decreased by miR-126 overexpression. **g** PLK-4, Ki-67, ATR and CHEK1 staining of tumor tissues from mice inoculated with mock and miR-126 infected SMMC-7721 cells Mean ± SD, *P < 0.05, **P < 0.01, unpaired Student’s *t* test or one-way ANNOVA followed by multiple *t* test. Scale bars = 100 μm. **h** Schematic representation showing miR-126/PLK-4 axis mediated aggressive behaviors in HCC through activating ATR/CHEK1 signaling pathway

Next, we further investigated the effect of miR-126 on HCC proliferation using a nude mice xenograft tumor model. Nude BALB/c male mice were s.c. xenograft with SMMC-7721 stable cell line expressing miR-126. Overexpression of miR-126 was observed in the group of lenti-miR-126 (Fig. 7a) and dramatically reduced tumor cell size and weight 28 days after inoculation compared with control SMMC-7721 cell group (Fig. 7b–d). In addition, ex vivo imaging clearly revealed that xenograft tumors grown from cells overexpressing miR-126 had smaller mean luciferase signal than the tumors developed from control cells (Fig. 7e, f). In addition, IHC analysis revealed that the tumor tissues of the miR-126 overexpression group displayed much weaker staining of PLK-4, Ki-67 (Fig. 7g). Importantly, ATR and CHEK1 expression were both inhibited in the tumor tissues of miR-126 overexpression group mice (Fig. 7g). Taken together, these results indicated that miR-126 could suppress HCC proliferation in vivo, at least in part, through regulating PLK-4/ATR/CHEK1 pathway.

## Discussion

Several studies have indicated that miR-126 played an important role in the progression of HCC. For instance, miR-126 served as a tumor suppressor in HCC progression through targeting sex-determining region Y-box 2 (Sox2)^28^. Downregulation of miR-126 played an important role in HCC metastasis and predicted poor prognosis for HCC patients^14^. Consistent with these reports, in this study, we further confirmed the downregulated miR-126 was positively associated with tumor stage, metastasis, recurrence and overall survival rate in a relative large scale of HCC patients’ cohorts. Additionally, subsequent functional studies showed that overexpression of miR-126 not only significantly reduced cell capacity of proliferation but also the ability of migration and invasion, as well as promoted cell apoptosis compared to control group in vitro. Furthermore, in vivo experiments, we found that overexpression of miR-126 significantly tumor proliferation. Our study demonstrates that miR-126 is frequently downregulated and functions as a tumor suppressor in human HCC.

It has been widely confirmed that miRNAs exert functions by binding to the their target genes^29^. Previous studies have shown that miR-126 serves as a tumor suppressor via targeting several genes involved in diverse tumors, including VEGF^12^, EGFL7^30^ and CXCR4^31^ and so on. In the current study, we newly identified PLK-4 as a directly target of miR-126 in HCC. The important role of PLK-4 in cell proliferation and tumorigenesis has been well characterized. Studies by Tian et al. showed that overexpression of PLK4 in neuroblastoma cells promoted EMT through the PI3K/Akt signaling pathway^32^. PLK-4 deletion caused death of breast cancer cells and inhibited breast cancer xenograft growth^33^. However, PLK-4 was previously show to be decreased in HCC based on limited number of clinical samples in single center^16,34^. In present study, we found the commonly upregulated PLK-4 expression in TCGA Pan-cancer analysis. Additionally, we validated the upregulated expression pattern of PLK-4 at both mRNA and protein level in a relative large scale of HCC tissues from multi-center. Meanwhile, high PLK-4 expression was significantly correlated with TNM stage, vascular invasion and poor OS, indicating the functional oncogenic role of PLK-4 in the progression of HCC. Contradictory results about PLK-4 expression in HCC may be due to the ethnic differences or statistical deviation caused by limited sample size of study.

Thereafter, using a series of in vitro and in vivo assays, we investigated whether PLK-4 mediated the suppressive effects of miR-126 in proliferation and metastasis of HCC cells. Our findings exhibited that PLK-4 silencing could significantly inhibit HCC cell proliferation in vitro and in vivo, and promote apoptosis. In addition, the suppressive effects of PLK-4 silencing on proliferation in HCC cells could be reversed by transfection with PLK-4 plasmid or transfection with miR-126 inhibitor. Although miR-126 expression has been reported previously in other human cancers, ours is the first study that provides a novel and comprehensive insight into the functional role of miR-126 in HCC by directly targeting PLK-4.

Subsequently, we performed a bioinformatics and carried out pathway enrichment analysis for PLK-4-associated signal pathways and finally focused on ATR/CHK1 pathway signal pathways due to its consentaneous function in cell cycle. Mounting evidence suggests that ATR/CHK1 pathway, frequently upregulated in human neoplasm, may promote tumor growth^35,36^. ATR is a serine/threonine protein kinase, which is dispensable to regulate DDR in cells with DNA crosslinks^37^. CHEK1 is important to mediate DNA damage repair by activating repair factors such as Rad51, FANCE and PCNA^38^. In cells with DNA crosslink, ATR activates CHEK1 by phosphorylation of at Ser-317 and Ser-345^22^. In our study, we found that both ATR and CHEK1 protein expression levels were upregulated in HCC tissues. Additionally, after PLK-4 knockdown, the expression levels of ATR and CHEK1 were both significantly decreased and there was a strong relationship among PLK-4, ATR and CHEK1. We also validated that PLK4 could interacted with ATR directly through CoIP assay and forced change of miR-126 expression not only led to PLK-4 but also ATR and CHEK1 dysregulation. Thus, ATR/CHEK1 pathway was a critical downstream molecule of PLK-4 in HCC cells. More importantly, high ATR and CHEK1 were significantly associated with poor prognosis for HCC patients from TCGA LIHC database. Taken together, these results suggested that PLK-4 served as an oncogene probably by activating ATR/CHEK1 pathway in HCC (Fig. 7h).

## Conclusions

In conclusion, we demonstrate the clinical and biological significance of miR-126/PLK-4 axis in HCC, and discovered the downstream effector ATR/CHEK1 pathway. The miR-126/PLK-4 axis inhibits tumor tumorigenesis and progression mainly through regulating proliferation and cell cycle. In this process, the ATR/CHEK1 pathway is significantly affected. These observations provide new evidence for interplay between cancer-associated signaling pathways and cell cycle. Targeting this newly identified regulatory signaling pathway provides therapeutic opportunities for aggressive liver cancer.

## Material and methods

### Patients and specimens

Fresh tissue from 75 patients (ZZU cohort 1) who were diagnosed as HCC in the First Affiliated Hospital of Zhengzhou University from 2009 to 2012 was selected for this study. Tissue microarrays (TMA) containing 396 paired paraffin embedding HCC specimens (341 with available follow-up data) and corresponding non-tumor tissues obtained from the First Affiliated Hospital of Zhengzhou University from 2011 to 2015 (ZZU cohort 2), were constructed using diameter of 1.5-mm cores. All the experiments were approved by the Institutional Review Board of the First Affiliated Hospital of Zhengzhou University.

### Cell culture

Normal and HCC cell lines were purchased from ATCC (Manassas, USA) or Sibcb (Shanghai, China). Cells were cultured in DMEM supplied with 10% fatal bovine serum, and 100 U/ml penicillin/streptomycin. Cells were cultured in a CO_2_ incubator at 37 °C. miR-126 mimics or anti-miR-126 oligonucleotides (Ambion, Austin, USA) were transfected using Lipofectamine 2000 reagent (Invitrogen, Carlsbad, USA) following the manufacturer’s protocol. For miR-126 overexpression, the cDNA was cloned into the BLOCK-iT™ Lentiviral Pol II miR RNAi Expression vector (Invitrogen, Carlsbad, USA). The SMMC-7721 cells with stable overexpression of miR-126 were transfected using lenti-virus infection. Cells polyclonal derivatives with hygromycin selection were used to avoid clonal variations in functional assays. The cells used in this study were listed in Supplementary Table 3.

### The gene set enrichment analysis (GSEA)

GSEA was used to determine which gene sets was associated with PLK4, ATR, CHEK1 expression in TCGA data set. The expression profiles of 377 samples from TCGA data set was grouped two classes according to gene expression. GSEA v2.0 was used to determine whether the members of the gene sets from the MSigDB database v4.0 are randomly distributed at the top or bottom of the ranking. The significance threshold was set at *P* < 0.05.

### Total RNA isolation and quantitative real-time PCR (qPCR)

Total RNA was isolated using TRIzol reagent (Life Technologies, Garlsbad, USA), and cDNA was generated using the SuperScript III First-Strand Synthesis System (Life Technologies, Garlsbad, USA). qPCR was performed using a Quantstudio 6 system (ABI, Foster City, USA) with powerup SYBR Green kit (ABI, Foster City, USA). The relative expression levels of the target genes were normalized to the expression level of GAPDH or U6. The data analyses were performed using the 2^−ΔΔCt^ method. The primer sequences are provided in Supplementary Table 4.

### Western blot analysis

Cells were collected at 48 h after transfection. Samples were probed with PLK-4, ATR, CHEK1 or β-actin monoclonal antibody. Goat anti-mouse HRP antibodies were obtained from Zhongshan Jinqiao Company, Beijing. ECL detection system (Millipore, Bedford, MA, USA) is used to assess proteins expression. The antibodies used in this study are provided in Supplementary Table 5.

### Co-immunoprecipitation (CoIP)

Proteins were extracted from the transfected cells. 30 µl sepharose beads (Santa Cruz Biotechnology, Inc.) and cell lysates were mixed to a volume of 400 µl. The cell lysates obtained from the cells were precleared by incubation for 2 h at 4 °C on a shaker. The clear supernatant was incubated overnight with GFP, anti-GFP (ProteinTech Group, Wuhan) or anti-FLAG antibody (ProteinTech Group, Wuhan). The samples were then washed with 0.5% NP40 cell lysate three times. Immobilized protein complexes were eluted by denaturation in 2 × SDS sample buffer at 95 °C for 10 min for subsequent western blotting.

### Collection of liver cancer microarray data sets

A total of 375 liver cancer data and 50 non-tumor data, with at least 10 years of follow-up, from The Cancer Genome Atlas (TCGA, https://tcga-data.nci. nih.gov/tcga/, updated to the end of 31 December 2016) database, were enrolled in this study for gene expression analysis and patients’ survival analyses.

Sixteen liver cancer mRNA and four miRNA microarray data sets that have accompanying scientific publications were assembled via the Gene Expression Omnibus (GEO) of the National Center for Biotechnology Information (NCBI). The BRB-array tools were used to directly determine the differentially expressed genes between healthy or adjacent liver tissue control samples and HCC samples in each data set. The detailed information was showed in Supplementary Table 6.

### Cell proliferation assay

For MTT assay, Hep-3b and SMMC7721 cells (5 × 10^3^ in 100 μL) were seeded into a 96-well plate. Each group included three repeated wells. At 1, 2, 3, 4 and 5 days after incubation, proliferation was assessed by MTT solution (Promega, Madison, WI, USA) using the standard protocol. The absorbance at a wavelength of 490 nm (A490) was measured on a SPECTRAmax Microplate Spectrophotometer from Molecular Devices (Sunnyvale, CA). For EDU assay, the DNA synthesized rate was assayed with EdU assay kit (Ribobio, Guangzhou, China) according to the manufacturer’s instructions. For the colony formation assay, 1000 cells were plated in 6-well plates. 2 weeks later, the cells were fixed with formaldehyde f and stained with crystal violet. The colony (defined as more than 50 cells/colony) number was counted using an optical microscope.

### Wound healing and cell apoptosis assay

Hep3B and SMMC7721 cells (5 × 10^6^) were cultured into each well of 6-well plates after transfection. A sterile micropipette tip was used to scratch the cells. Three wounds were made for each group. The scratched wound was photographed and the distance was measured every 24 h. For cell apoptosis, FACS analysis for apoptosis was done using PE Annexin V apoptosis detection kits (BD Pharmingen, USA) after 48-hour transfection according to the manufacturer’s protocol.

### Transmembrane invasion assay

Human hepatocellular carcinoma Hep3B and SMMC-7721 cells were transfected with miR-126 mimics (5′-UCGUACCGUGAGUAAUAAUGCG-3′, Genepharma, China) or scramble (5′-UUCUCCGAACGUGUCACGUTT-3′). After 48 h, 5 × 10^6^/mL cells were transferred into the upper chamber of the Millicell inserts pre-coated with 20 µg Matrigel and pre-incubated 1 h to reconstitute a basement membrane (Millipore, USA) in a serum-free DMEM. DMEM containing 10% fetal bovine serum was added to the lower chamber. Assay was allowed to proceed for 24 h in a CO_2_ incubator at 37 °C. After 24 h incubation, the uninvaded cells were gently removed from the inner part of the insert. The cells that had invaded through the membrane were fixed with methanol and stained with 0.5% crystal violet.

### Construction of the HCC tissue microarray (TMA)

A retrospective study of 75 HCC patients was detected for qPCR analysis. The information of clinico-pathological parameters was displayed in Supplementary Table 2. Another cohort of 369 HCC patients was identifed for TMA immunohistochemical staining. Clinico-pathological features of our cohort as shown in Supplementary Table 3. From one paraffn block of each tumor, a 5-μm diameter tissue section was cut and mounted consecutively on the recipient master blocks. The TMA was constructed by the Tissue Microarray and Imaging Core at the First Affliated Hospital of Zhengzhou University

### Immunohistochemical (IHC) staining on TMA

For H&E staining, sections were deparaffinized and rehydrated. Sections were then stained in Mayer hematoxylin solution for 8 min. Sections were then washed in warm running tap water for 10 min, and rinsed in distilled water and 95% alcohol for 10 dips. Counterstain in eosin-phloxine Y solution for 1 min. Dehydrate sections through 95% alcohol, 2 times of absolute alcohol, 5 min each time. Clear sections through 2 times of xylene, 5 min each. Mount sections with xylene based mounting medium. For PLK-4, ATR, CHEK1 and Ki67 staining, antigen-retrieved sections were washed 2X with PBS and 1X PBST (0.1% v/v). After slides were blocked with 3% BSA for 1 h at room temperature, slides were added antibodies (PLK-4 1:100 dilution, Ki-67 1:100 dilution, Millipore, USA) and incubated for 1 h at room temperature. Detection was carried out using the HRP-DAB system (Millipore, USA). The antibodies used in this study are provided in Supplementary Table 3.

### Luciferase activity assay

On the basis of Platinum Taq DNA polymerase (Life Technologies), wild-type or mutant fragments of the PLK-4 3′UTR containing the predicted miR-126-binding site were amplified. In the pMIR-REPORT miRNA expression reporter vector (Life Technologies), the amplified PCR products were cloned. As a reference control using Lipofectamine 2000 (Life Technologies), the cells were co-transfected with 20 nM pre-miR, 100 ng pMIR-REPORT vector (Firefly luciferase), and 50 ng pRL-SV40 vector (Renilla luciferase, Promega). The relative luciferase activity was calculated by normalizing the Fireflyluminescence to the Renilla luminescence at 24 h post-transfection according to the manufacturer’s instructions.

### Animals

Male nude mice (product code:403, Beijing Vital River Laboratory Animal Technology Co., Ltd, China) were kept in a controlled environment (12 h light–dark cycle, 25 °C, 60–70% humidity) in animal facility of the First Affiliated Hospital of Zhengzhou University. For mice xenograft tumor model, 6- to 8-weeks-old male mice were used. SMMC-7721 cells (approximately 3.0 × 10^6^) transfected with lenti-miR-126 or lenti-MOCK were subcutaneously (s.c.) injected into the lower flank of the mice. The tumor volume was calculated by the formula: Volume = (width)^2^ × length / 2. Mice were photographed with an IVIS@ Lumina II system (Caliper Life Sciences, Hopkinton, MA) 10 min after an intraperitoneal injection of 4.0 mg of luciferin (Gold Biotechnology, Inc., St. Louis, MO) in 50 μl of saline. After 6 weeks, tumors were surgically removed and weighed. All research involving animals complied with protocols approved by the Animal Care and Use Committee of the First Affiliated Hospital of Zhengzhou University (Reference number: 2015–329).

### Statistical analysis

All the data were presented as Mean ± SD unless indicated otherwise. The Student’s *t* test was used to calculate the difference between two groups of data, while one-way ANOVA followed by Bonferroni correction was used to calculate the difference among the three groups. Clinico-pathological variables were analyzed by *Χ*^2^ tests. Kaplan–Meier curves and the log-rank tests were used to analyze the overall survival (OS) of HCC patients and mice tumor xenograft results. Univariate and multivariate Cox regression analysis were conducted to identify the independent factors. All the statistical analyses were performed using the SPSS 20.0 software program (SPSS Inc., Chicago, USA). *P* < 0.05 is considered to be statistical significance.

### Disclaimer

The funding body had no role in the design of the study, in the collection, analysis, and interpretation of the data, or in the manuscript writing.

## Electronic supplementary material

Supplementary Figure a-c

Supplementary Figure 1

Supplementary Figure 2

Supplementary table 1-6

## References

[1] Mcglynn KA, Petrick JL, London WT (2015). Global epidemiology of hepatocellular carcinoma: an emphasis on demographic and regional variability. Clin. Liver. Dis..

[2] Elserag HB, Kanwal F (2014). Epidemiology of hepatocellular carcinoma in the United States: where are we? Where do we go?. Hepatology.

[3] Jordi B, Morris S (2011). Management of hepatocellular carcinoma: An update. J. Gastrointest. Surg..

[4] Müller C (2006). Hepatocellular carcinoma – Rising incidence, changing therapeutic strategies. Wien. Med. Wochenschr..

[5] Cortez MA, Calin GA (2009). MicroRNA identification in plasma and serum: a new tool to diagnose and monitor diseases. Expert. Opin. Biol. Ther..

[6] Ambros V (2004). The functions of animal microRNAs. Nature.

[7] Choi E, Hwang KC (2013). MicroRNAs as novel regulators of stem cell fate. World J. Stem Cells.

[8] Zhuang LK (2016). MicroRNA-92b promotes hepatocellular carcinoma progression by targeting Smad7 and is mediated by long non-coding RNA XIST. Cell Death Dis..

[9] Ghosh A (2017). MiRNA199a-3p suppresses tumor growth, migration, invasion and angiogenesis in hepatocellular carcinoma by targeting VEGFA, VEGFR1, VEGFR2, HGF and MMP2. Cell Death Dis..

[10] Chao Y (2017). miR-1301 inhibits hepatocellular carcinoma cell migration, invasion, and angiogenesis by decreasing Wnt/β-catenin signaling through targeting BCL9. Cell Death Dis..

[11] Feng R (2010). miR-126 functions as a tumour suppressor in human gastric cancer. Cancer Lett..

[12] Liu B, Peng XC, Zheng XL, Wang J, Qin YW (2009). MiR-126 restoration down-regulate VEGF and inhibit the growth of lung cancer cell lines in vitro and in vivo. Lung Cancer.

[13] Song L, Xie X, Shaojie YU, Peng F, Peng L (2015). MicroRNA-126 inhibits proliferation and metastasis by targeting pik3r2 in prostate cancer. Mol. Med. Rep..

[14] Chen H (2013). Decreased expression of miR-126 correlates with metastatic recurrence of hepatocellular carcinoma. Clin. Exp. Metastas-..

[15] Lowery DM, Lim D, Yaffe MB (2005). Structure and function of Polo-like kinases. Oncogene.

[16] Pellegrino R (2010). Oncogenic and tumor suppressive roles of polo-like kinases in human hepatocellular carcinoma. Hepatology.

[17] Habedanck R, Stierhof YD, Wilkinson CJ, Nigg EA (2005). The Polo kinase Plk4 functions in centriole duplication. Nat. Cell Biol..

[18] Ko MA (2005). Plk4 haploinsufficiency causes mitotic infidelity and carcinogenesis. Nat. Genet..

[19] Shinmura K (2014). PLK4 overexpression and its effect on centrosome regulation and chromosome stability in human gastric cancer. Mol. Biol. Rep..

[20] Korzeniewski N, Hohenfellner M, Duensing S (2012). CAND1 promotes PLK4-mediated centriole overduplication and is frequently disrupted in prostate cancer. Neoplasia.

[21] Smith J, Tho LM, Xu N, Gillespie DA (2010). The ATM-Chk2 and ATR-Chk1 pathways in DNA damage signaling and cancer. Adv. Cancer Res..

[22] Flynn RL, Zou L (2011). ATR: a master conductor of cellular responses to DNA replication stress. Trends Biochem. Sci..

[23] Abdel-Fatah TMA (2015). Untangling the ATR-CHEK1 network for prognostication, prediction and therapeutic target validation in breast cancer. Mol. Oncol..

[24] Parikh RA (2013). Upregulation of the ATR-CHEK1 pathway in oral squamous cell carcinomas. Genes Chromosomes Cancer.

[25] Okazaki T (2007). Single nucleotide polymorphism of ATM and CHEK-1 genes are associated with survival of pancreatic cancer. Cancer Res..

[26] Bartel D (2004). MicroRNAs: genomics, biogenesis, mechanism, and function. Cell.

[27] Rebouissou S (2017). Proliferation markers are associated with MET expression in hepatocellular carcinoma and predict tivantinib sensitivity in vitro. Clin. Cancer Res..

[28] Zhao C, Li Y, Zhang M, Yang Y, Chang L (2015). miR-126 inhibits cell proliferation and induces cell apoptosis of hepatocellular carcinoma cells partially by targeting Sox2. Hum. Cell.

[29] Moss EG (2002). MicroRNAs: hidden in the genome. Curr. Biol..

[30] Yanqin (2010). miR-126 inhibits non-small cell lung cancer cells proliferation by targeting EGFL7. Biochem. Biophys. Res. Commun..

[31] Z L (2013). Expression of miR-126 suppresses migration and invasion of colon cancer cells by targeting CXCR4. Mol. Cell. Biochem..

[32] Tian X (2018). Polo-like kinase 4 mediates epithelial-mesenchymal transition in neuroblastoma via PI3K/Akt signaling pathway. Cell Death Dis..

[33] Li Z (2016). Expression of Polo-like kinase 4(PLK4) in breast cancer and its response to Taxane-based neoadjuvant chemotherapy. J. Cancer.

[34] Liu L (2012). Downregulation of Polo-like kinase 4 in hepatocellular carcinoma associates with poor prognosis. PLoS ONE.

[35] Verlinden L (2007). The E2F-regulated gene Chk1 is highly expressed in triple-negative estrogen receptor−/progesterone receptor−/HER-2− breast carcinomas. Cancer Res..

[36] Albiges L (2014). Chk1 as a new therapeutic target in triple-negative breast cancer. Breast.

[37] Cimprich KA, Cortez D (2008). ATR: an essential regulator of genome integrity. Nat. Rev. Mol. Cell Biol..

[38] Zhang Y, Hunter T (2013). Roles of Chk1 in cell biology and cancer therapy. Int. J. Cancer.

