# Supplementary material for: MiR-126 negatively regulates PLK-4 to impact the development of hepatocellular carcinoma via ATR/CHEK1 pathway
Source: Cell Death Dis. 2018 Oct 12;9(10):1045. doi: 10.1038/s41419-018-1020-0 (PMC6185973; doi:10.1038/s41419-018-1020-0)
Supplement: Supplementary file 1 — Supplementary Figure a-c [file 41419_2018_1020_MOESM1_ESM.doc]

**Figure S1.** (**a-c**) The correlation among PLK-4, ATR and CHEK1 expression were analyzed by pearson analysis. (**d, e**) The correlation between Ki-67 and ATR, CHEK1 expression were analyzed by pearson analysis. (**f, g**) High ATR and CHEK1 expression was significantly associated with poor DFS in TCGA-LIHC cohort.

**Figure S2.** (**a**) Differentially expressed mRNAs were analyzed based on PLK-4 expression in TCGA-LIHC cohort. (**b, c**) GO and KEGG pathway enrichment analysis were performed based on PLK-4 expression in TCGA-LIHC cohort. (**d, e**) The Gene Set Enrichment Analysis plot indicated a consistent correlation between PLK-4, ATR and CHEK1 expression and DNA replication and mismatch repair.
