# Supplementary figures and images for: MiR-126 negatively regulates PLK-4 to impact the development of hepatocellular carcinoma via ATR/CHEK1 pathway
Source: Cell Death Dis. 2018 Oct 12;9(10):1045. doi: 10.1038/s41419-018-1020-0 (PMC6185973; doi:10.1038/s41419-018-1020-0)

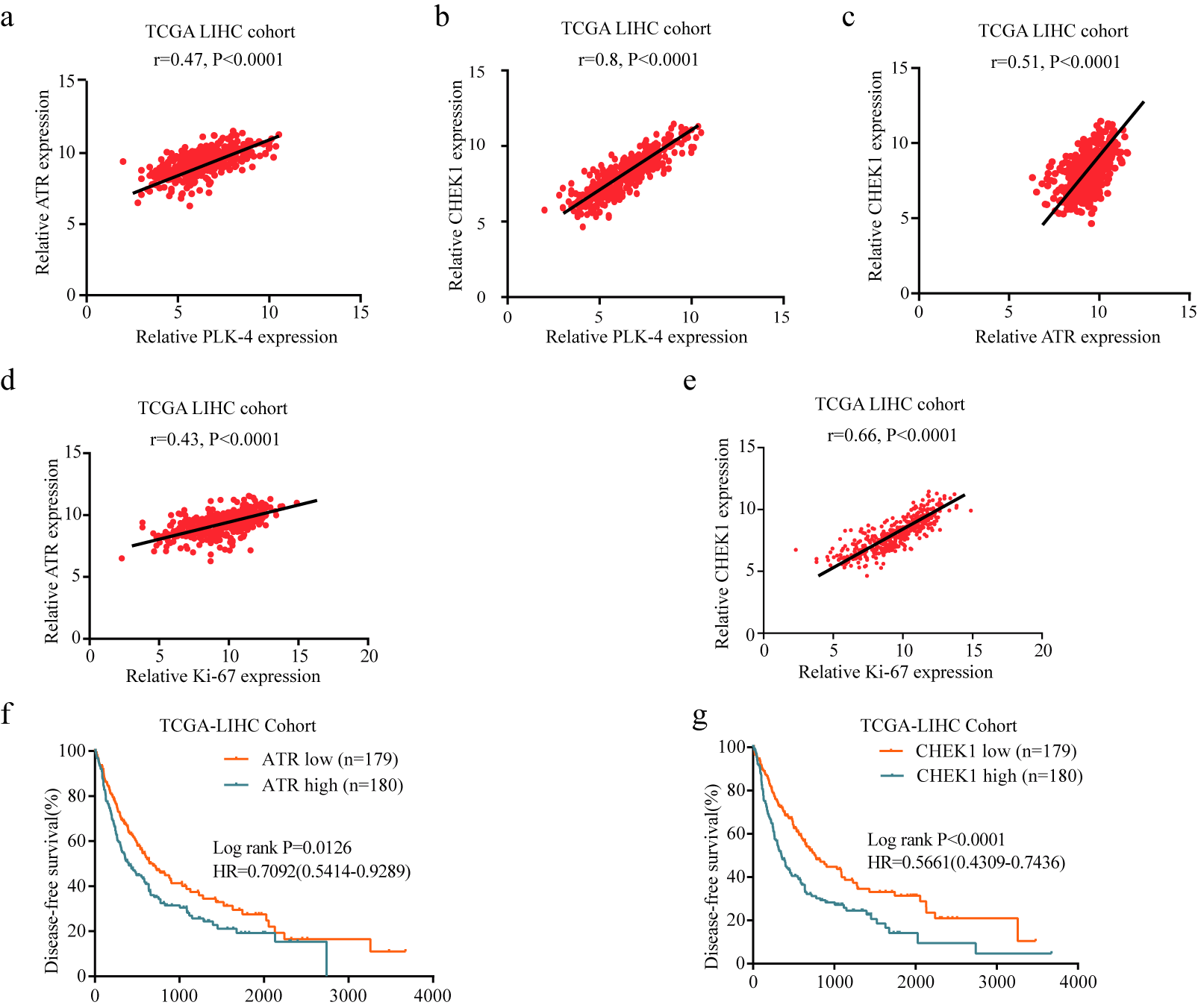

Supplement: Supplementary file 2 — Supplementary Figure 1 [file 41419_2018_1020_MOESM2_ESM.tif]

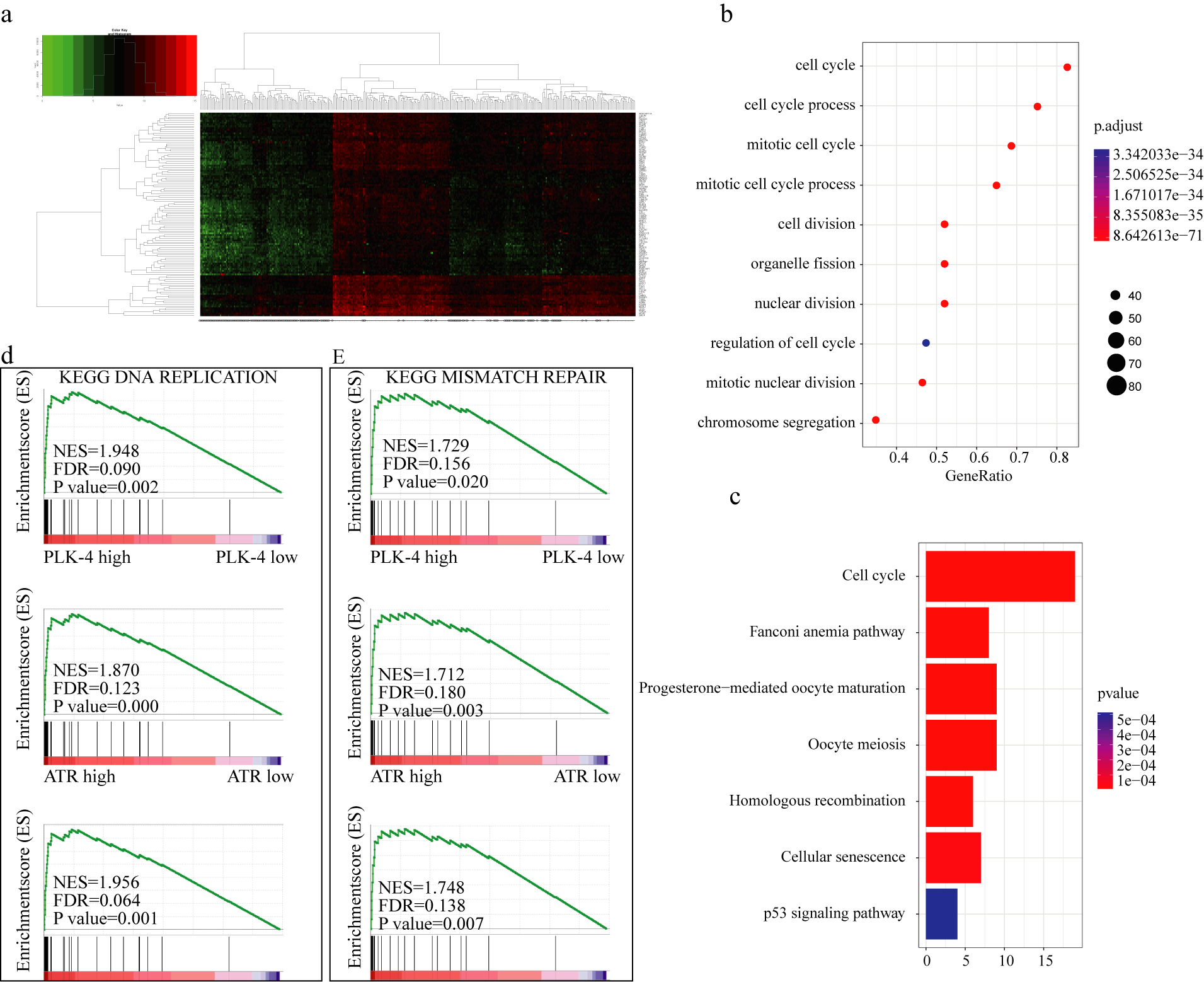

Supplement: Supplementary file 3 — Supplementary Figure 2 [file 41419_2018_1020_MOESM3_ESM.tif]
