# Supplementary material for: MiR-126 negatively regulates PLK-4 to impact the development of hepatocellular carcinoma via ATR/CHEK1 pathway
Source: Cell Death Dis. 2018 Oct 12;9(10):1045. doi: 10.1038/s41419-018-1020-0 (PMC6185973; doi:10.1038/s41419-018-1020-0)
Supplement: Supplementary file 4 — Supplementary table 1-6 [file 41419_2018_1020_MOESM4_ESM.docx]

**Supplementary Table 1.** **Association of miR-126 expression and clinical features in TCGA cohort**

| **Variables** | **Clinicopathological features** | **miR-126** | | ***P-value*** |
| --- | --- | --- | --- | --- |
|  |  | **Low (n=188)** | **High(n=187)** |  |
| Age (years) | ≤59 | 96 | 91 | 0.605 |
|  | >59 | 92 | 96 |  |
| Gender | Male | 61 | 59 | 0.825 |
|  | Female | 127 | 128 |  |
| TNM stage | Stage I and II | 115 | 133 | 0.131 |
|  | Stage III and IV | 50 | 37 |  |
|  | unknown | 23 | 17 |  |
| Liver Cirrhosis | Positive | 24 | 44 | **0.014** |
|  | Negative | 70 | 69 |  |
|  | unknown | 94 | 74 |  |
| Serum AFP(ng/mL) | ≤20 | 61 | 85 | **0.037** |
|  | >20 | 67 | 57 |  |
|  | unknown | 60 | 45 |  |

Bold values indicate statistical significance, *P* < 0.05

**Supplementary Table 2.** **Association of miR-126 expression and clinical features in ZZU cohort 1**

| Clinicopathological features | | miR-126 expression | | | χ2 | P-value | Survival | | χ2 | P-value |
| --- | --- | --- | --- | --- | --- | --- | --- | --- | --- | --- |
|  |  | Low (n=37) | High (n=38) | |  |  | Live Dead  (n=36) (n=39) | |  |  |
| Age(years) | >61 | 22 | | 16 | 2.258 | 0.1328 | 17 | 21 | 0.328 | 0.566 |
|  | ≤61 | 15 | | 22 |  |  | 19 | 18 |  |  |
| Gender | Male | 24 | | 19 | 1.387 | 0.238 | 23 | 20 | 1.216 | 0.270 |
|  | Female | 13 | | 19 |  |  | 13 | 19 |  |  |
| Aetiology | Viral | 30 | | 32 | 2.142 | 0.343 | 28 | 34 | 1.281 | 0.527 |
|  | Alcohol | 2 | | 0 |  |  | 1 | 1 |  |  |
|  | other | 5 | | 6 |  |  | 7 | 4 |  |  |
| TNM stage | Stage I and II | 15 | | 25 | 4.801 | 0.028 | 24 | 16 | 4.945 | **0.0261** |
|  | Stage III and IV | 22 | | 13 |  |  | 12 | 23 |  |  |
| AFP | ≤20 | 12 | | 14 | 0.160 | 0.688 | 15 | 11 | 1.497 | 0.221 |
|  | >20 | 25 | | 24 |  |  | 21 | 28 |  |  |
| Cirrhosis | Absent | 13 | | 9 | 1.185 | 0.276 | 14 | 8 | 3.049 | **0.008** |
|  | Present | 24 | | 29 |  |  | 22 | 31 |  |  |
| Metastasis  Or Recurrence | Absent | 13 | | 25 | 7.047 | 0.0079 | 27 | 11 | 14.983 | **0.0001** |
|  | Present | 24 | | 13 |  |  | 9 | 27 |  |  |
| miR-126  expression | Low | - | | - |  | - | 13 | 24 | 4.842 | **0.0271** |
|  | High | - | | - |  | - | 23 | 15 |  |  |

Bold values indicate statistical significance, *P* < 0.05

**Supplementary Table 3.** Cell lines used in this study

| **Cell lines** | **Type** | **Sourse** |
| --- | --- | --- |
| Chang liver | Normal liver cell | ATCC,USA |
| LO2 | Normal liver cell | Sibcb, China |
| HepG2 | Liver cancer cell | ATCC,USA |
| SMMC-7721 | Liver cancer cell | Sibcb, China |
| SK-Hep-3B | Liver cancer cell | Sibcb, China |
| MHCC97-H | Liver cancer cell | Sibcb, China |
| HCC-LM3 | Liver cancer cell | Sibcb, China |
| Bel-7402 | Liver cancer cell | Sibcb, China |

**Supplementary Table 4.** Primer sequence used in this study

| Name | Direction | Primer(5’-3’) |
| --- | --- | --- |
| miR-126 | Forward | 5'-GTCGTATCCAGTGCA GGGTCCGAG-3' |
|  | Reverse | 5'-GTATTCGCACTGGAT ACGAC-3' |
| U6 snRNA | Forward | 5'-CTCGCTTCGGCAGCA CA-3' |
|  | Reverse | 5'-AACGCTTCACGAATTTGCGT-3' |
| PLK4 | Forward | 5'-TCCAAGAGGCAGAAGAAAGACC-3' |
|  | Reverse | 5'-GCTGAGTGACATCGTTCCATTG-3' |
| GAPDH | Forward | 5'-GTCTCCTCTGACTTCAACAGCG-3' |
|  | Reverse | 5'-ACCACCCTGTTGCTGTAGCCAA-3' |

**Supplementary Table 5.** Information on antibodies used for the correlation analysis

| Antibody | WB | IHC | Specificity | Company |
| --- | --- | --- | --- | --- |
| β-actin | 1:2000 | / | Mouse monoclonal | Sigma.USA |
| PLK-4 | 1:1000 | 1:100 | Mouse monoclonal | Millipore, USA |
| ATR | 1:1000 | 1:100 | Mouse monoclonal | Millipore, USA |
| CHEK1 | 1:1000 | 1:100 | Mouse monoclonal | Millipore, USA |
| Ki-67 | / | 1:100 | Mouse monoclonal | Millipore, USA |

**Supplementary Table 6. HCC expression profile cohorts used in this study**

| Cohort ID | Platform | Number of samples | | Public year | Country |
| --- | --- | --- | --- | --- | --- |
|  |  | Non-tumor | Tumor |  |  |
| TCGA | Illumina | 50 | 374 | 2009 | USA |
| GSE10143 | DASL | 307 | 80 | 2008 | USA |
| GSE14520 | Affymetrix | 220 | 225 | 2010 | USA |
| GSE25097 | Affymetrix | 289 | 268 | 2011 | USA |
| GSE36376 | Illumina | 193 | 240 | 2012 | South Korea |
| GSE39791 | Illumina | 72 | 72 | 2014 | USA |
| GSE45436 | Affymetrix | 41 | 93 | 2014 | Taiwan China |
| GSE54236 | Agilent | 80 | 81 | 2014 | Italy |
| GSE57957 | Illumina | 39 | 39 | 2014 | Singapore |
| GSE60502 | Affymetrix | 18 | 18 | 2015 | Taiwan China |
| GSE62232 | Affymetrix | 10 | 81 | 2014 | France |
| GSE64041 | Affymetrix | 60 | 60 | 2016 | Switzerland |
| GSE76427 | Illumina | 52 | 115 | 2017 | Singapore |
| GSE77314 | Illumina | 50 | 50 | 2016 | China |
| GSE84598 | Illumina | 44 | 22 | 2017 | Germany |
| GSE102083 | Affymetrix | 105 | 152 | 2018 | Japan |
| GSE10694 | CapitalBio | 88 | 78 | 2008 | China |
| GSE30297 | Affymetrix | 36 | 61 | 2011 | USA |
| GSE40744 | Affymetrix | 67 | 9 | 2012 | USA |
| GSE67138 | Affymetrix | 34 | 23 | 2015 | USA |
| Total |  | **1855** | **2141** |  |  |
